# Suitability of new drugs registered in Brazil from 2003 to 2013 for pediatric age groups

**DOI:** 10.31744/einstein_journal/2018AO4354

**Published:** 2018-10-30

**Authors:** Jaqueline Cristina da Silveira Xavier e Castro, Stephanie Ferreira Botelho, Taisa Roberta Lopes Machado, Maria Auxiliadora Parreiras Martins, Liliana Batista Vieira, Adriano Max Moreira Reis

**Affiliations:** 1 Faculdade de Farmácia, Universidade Federal de Minas Gerais, Belo Horizonte, MG, Brazil.; 2 Faculdade de Ciências Farmacêuticas, Universidade Federal de Alfenas, Alfenas, MG, Brazil.

**Keywords:** Reference drugs, Drug approval, Drug therapy, Dosage forms, Child, Medicamentos de referência, Aprovação de drogas, Tratamento farmacológico, Formas de dosagem, Criança

## Abstract

**Objective:**

To analyze suitability of new drugs registered in Brazil from 2003 to 2013 for pediatric age groups.

**Methods:**

A descriptive study of drugs with pediatric indication included in a retrospective cohort of new drugs registered in Brazil. The evaluation of drug suitability for the pediatric age group was performed using the following criteria: suitability of dosage form and capacity to deliver the recommended dose. The drugs were considered adequate for the pediatric age groups when they met both criteria. The statistical analysis included calculation of frequencies and proportions.

**Results:**

Suitability due to the drug capacity to deliver the recommended dose was greater than 80% across all age groups. Regarding suitability of the dosage form, we identified that the older the age group, the greater suitability for pediatric use. Concerning the drugs presented in solid dosage form, we showed that half were classified as inadequate for one or more pediatric age groups to whom they were indicated. The adequacy of drugs to the pediatric age group was 64.3% for preschool children, 66.7% for full-term newborns, 66.7% for premature newborns, and over 70% for other age groups.

**Conclusion:**

Drugs for children aged under 6 years were less often adequate, considering the dosage form and capacity to provide the recommended dose. The availability and proportional suitability of medicines for pediatric use are greater for older age groups, according to age groups the drug is registered for.

## RESUMO

**Objetivo:**

Analisar a adequação às faixas etárias pediátricas dos medicamentos novos registrados no Brasil no período de 2003 a 2013.

**Métodos:**

Estudo descritivo dos medicamentos com indicação pediátrica incluídos em uma coorte retrospectiva de medicamentos novos registrados no Brasil. A avaliação da adequação do medicamento à faixa etária pediátrica foi realizada empregando os seguintes critérios: adequação da forma farmacêutica e capacidade de fornecer a dose recomendada. Os medicamentos foram considerados adequados às faixas etárias pediátricas quando preencheram os dois critérios. A análise estatística compreendeu cálculo de frequências e proporções.

**Resultados:**

A adequação devido à capacidade do medicamento fornecer a dose recomendada foi superior a 80% em todas as faixas etárias. Em relação à adequação da forma farmacêutica, identificou-se que quanto maior a faixa etária, maior a proporção de adequação para uso pediátrico. Em relação aos medicamentos que se apresentavam em formas farmacêuticas sólidas, evidenciou-se que metade foi classificada como inadequada para uma ou mais faixas etárias pediátricas para as quais estavam indicados. A adequação dos medicamentos à faixa etária pediátrica foi 64,3% para pré-escolares, 66,7% para recém-nascidos a termo, 66,7% para recém-nascidos prematuros e superior a 70% para as demais faixas etárias.

**Conclusão:**

Os medicamentos destinados às crianças menores de 6 anos apresentaram menor frequência de adequação, considerando a forma farmacêutica e a capacidade de fornecer a dose recomendada. A disponibilidade e a proporção de adequação dos medicamentos para uso pediátrico aumentam com a elevação da faixa etária para a qual o medicamento é registrado.

## INTRODUCTION

Children are not small adults, but rather a distinctive and heterogeneous group of patients, with specific needs regarding medications.^(^ ^1^ ^-^ ^3^ ^)^ Heterogeneity results from the changes that occur in childhood related to growth and development of organs and physiological systems, which influence pharmacokinetics and pharmacodynamics,^(^ ^1^ ^,^ ^2^ ^)^ as well as changes in motor and cognitive skills that interfere in the administration of medications.^(^ ^3^ ^)^ Thus, children have different needs than adults do when it comes to dosage, dosage form, and ease in administrating medications.^(^ ^1^ ^,^ ^3^ ^,^ ^4^ ^)^

In order to reach accuracy of the dose given, reduce medication errors, increase adherence with treatment, and improve therapeutic results in pediatrics, the availability of medications in dosage form and formulations adequate for children’s needs is vital.^(^ ^2^ ^)^

The development of medications as per the principles of a formulation centered on the patient^(^ ^5^ ^,^ ^6^ ^)^ and the appropriate age^(^ ^4^ ^)^ is a current tendency, to satisfy the specificities in pediatrics. A medication appropriate for the child presents with a formulation that provides variable release of the dose according to weight/height, acceptable palatability, and safety associated with the excipients, ease in swallowing, an adequate device for measuring the dose, and skill in administration compatible with the pediatric age range for which it is destined.^(^ ^4^ ^,^ ^5^ ^,^ ^7^ ^)^

In different countries, the number of medications registered for pediatric use is lower than that for adults, and unavailability is higher for younger children.^(^ ^8^ ^-^ ^11^ ^)^ In Brazil, there is also a lack of medications suitable for pediatric use.^(^ ^12^ ^)^

Due to the reduced number of medications appropriate for pediatric age ranges, the off-label or unlicensed use of medications is a problem that persists in various health care scenarios, including in Brazil, rising the risk of adverse reactions and therapeutic ineffectiveness.^(^ ^12^ ^)^

## OBJECTIVE

To analyze the suitability of dosage form of new drugs registered in Brazil, from 2003 to 2013, for pediatric age ranges.

## METHODS

This is a descriptive study of drugs with pediatric indications included in a retrospective cohort of new drugs registered in Brazil. The retrospective cohort includes new medications registered by the *Agência Nacional de Vigilância Sanitária* (ANVISA) [Health Surveillance Agency], between January 2003 and December 2013. The cohort was prepared by consulting the Drugs@FDA (https://www.accessdata.fda.gov/scripts/cder/daf/) database; the ANVISA publication section available in the *Diário Oficial da União* [Official Federal Gazette], and the review article ‘To market, to market’ published in the Annual Reports in Medicinal Chemistry. The detailed description of the preparation of the cohort was presented in a previous study.^(^ ^13^ ^)^

The medications that were still on the market were identified on the drug price list of January 2016, published by ANVISA.^(^ ^14^ ^)^ The electronic medication guide of ANVISA was searched to check if the new drugs included in the cohort and on the market in the country in 2016 had registration and pediatric indication at ANVISA.^(^ ^15^ ^)^ When the medication was not included in the guide, the package insert was requested, by e-mail, to the manufacturing industry.

Classification of the medications was done according to the third pharmacological therapeutic level of the Anatomical Therapeutic Chemical Code (ATC), of the World Health Organization (WHO).^(^ ^16^ ^)^

To characterize the pediatric indication in this investigation, the following age stratification was adopted: premature newborns (<37 weeks), full-term newborns (≥37 weeks and <28 days), infants (≥28 days and <2 years), preschool (≥2 and <6 years), school aged (≥6 and <12 years), and adolescents (≥12 years and <19 years).^(^ ^17^ ^)^

The assessment of the adequacy for the pediatric age range was performed taking into consideration the pharmaceutical presentations sold in Brazil in 2016, and the following criteria were used: suitability of the formulation and capacity to deliver the recommended dose. The medications were considered adequate for the pediatric age ranges when both criteria were met.

The evaluation of suitability of the dosage form for the pediatric age ranges was done using the criteria defined by the European Medicines Agency (EMA) in the document ‘Reflection paper: formulations of choice for the pediatric population’.^(^ ^18^ ^)^ The criteria were developed by using a matrix-based strategy with the following factors: age range stratified as per Willians et al.,^(^ ^17^ ^)^ dosage form, and route of administration, considering suitability in pediatrics. The suitability of the medication according to the matrix-based strategy was defined on a Likert-type scale. For children aged under 11 years, the scale is structured as (1) unsuitable, (2) suitable with problems, (3) suitable but not preferable, (4) good suitability, and (5) excellent suitability. For adolescents, the scale is: (1) unacceptable, (2) acceptable with restrictions, (3) acceptable, (4) preferred, and (5) dosage form of choice.^(^ ^18^ ^)^ In this investigation, the dosage form was classified as suitable for the age range for which the medication was registered, when it showed a score higher than 3, for children younger than 12 years, and over 2, for adolescents.

Also evaluated was the capacity of the medication, in the dosage form in which it is marketed, to deliver the dose recommended. This assessment was done as per Fontan et al.,^(^ ^19^ ^)^ who classifies as unsuitable the solid dosage form − there is a need for division in order to obtain the proper dose − and the liquid dosage form − there is a need to administer a volume of medication smaller than 1mL. All other dosage forms were considered suitable to deliver an exact dose necessary for the child. The evaluation of suitability was made considering the dose prescribed for children described in the package insert. For the medication whose dose was expressed in mg/kg, reference weights were used for each age range, according to the curves recommended by the WHO for the ideal weight of children, considering the lower limits of the age range.^(^ ^20^ ^)^

In the pharmaceutical specialty listings of the 46 medications with a pediatric indication, the presence of the following pharmaceutical excipients having a potential to harm children was identified: antioxidants (sulfites),^(^ ^21^ ^)^ solubilising agents (polysorbate 80^(^ ^22^ ^,^ ^23^ ^)^ and cyclodextrin),^(^ ^10^ ^)^ antimicrobial preservatives (parabens − propylparaben, ethylparaben, and methylparaben;^(^ ^22^ ^,^ ^23^ ^)^ benzoates − benzyl alcohol, benzoic acid, sodium benzoate,^(^ ^10^ ^,^ ^21^ ^-^ ^23^ ^)^ − and benzalkonium chloride),^(^ ^10^ ^,^ ^21^ ^-^ ^23^ ^)^ diluting agent (lactose),^(^ ^21^ ^,^ ^24^ ^)^ sweetening agents (aspartame,^(^ ^22^ ^,^ ^23^ ^)^ sorbitol,^(^ ^10^ ^,^ ^23^ ^)^ saccharine^(^ ^10^ ^,^ ^22^ ^)^), and solvents (ethanol and propylene glycol,^(^ ^10^ ^,^ ^22^ ^-^ ^24^ ^)^ and peanut oil).^(^ ^22^ ^)^

### Statistical analysis

The database was prepared on the Epidata 3.1 software. The statistical analysis included the calculation of frequencies and proportions, and used the Statistical Package for Social Sciences software, version 21.0.

## RESULTS

From January 2003 to December 2013, Brazil registered 159 new medications, and 25 had their sale interrupted in the country after registration. Thus, the cohort investigated included 134 new medications that were on the market in January 2016. Among the 134 new medications, 46 (34.3%) were identified as having pediatric indications in other countries, and 37 (27.6%) were registered for use in children of Brazil.

As to the ATC classification ( Table 1 ), the most frequent drugs belonged to the following groups: (A) digestive tract and metabolism (26.1%), (J) anti-infectious agents for systemic use (21.7%), (L) antineoplastic and immunomodulating agents (13%), and (R) respiratory system (10.9%).

**Table 1:** Anatomical Therapeutic Chemical Classification( ^16^ ) of the 46 pediatric medications identified in the cohort of new medication, registered from 2003 to 2013

ATC Classification		n (%)
Alimentary tract and metabolism		12 (26.1)
A02B	Drugs for peptic ulcer and gastroesophageal reflux disease: dexlansoprazole	1 (2.2)
A04A	Antiemetic and anti-nausea agents: aprepitant and palonosetron	2 (4.4)
A10A	Insulin and analogues: insulin detemir and insulin glulisine	2 (4.4)
A16A	Other alimentary tract and metabolism products: alglucosidase alfa, velaglucerase alfa, sapropterin, galsulfase, idursulfase, laronidase, and miglustate	7 (15.2)
Cardiovascular system		1 (1)
C10A	Lipid modifying agents: rosuvastatin	1 (2.2)
Anti-infectious agents for systemic use		10 (21.7)
J02A	Antifungal agents for systemic use: anidulafungin, micafungin, and posaconazole	3 (6.5)
J05A	Antivirals with direct action: enfuvirtide, entecavir, darunavir, etravirine, fosamprenavir, raltegravir potassium, and atazanavir	7 (15.2)
Antineoplastic and immunomodulating agents		6 (13.0)
L01X	Other antineoplastic agents: nimotuzumab	1 (2.2)
L04A	Immunosuppressants agents: abatacept, adalimumab, canakinumab, everolimus, and tocilizumab	5 (10.9)
Respiratory system		5 (10.9)
R01A	Decongestants and other nasal preparations for topical use: ciclesonide and fluticasone	2 (4.4)
R03D	Other systemic drugs for obstructive airway diseases: omalizumab	1 (2.1)
R06A	Anti-histamines for systemic use: bilastine and rupatadine	2 (4.4)
Nervous system		4 (8.8)
N05A	Antipsychotics: asenapine and paliperidone	2 (4.4)
N06A	Antidepressants: duloxetine	1 (2.2)
N06B	Psychostimulants, agents used for ADHD: lisdexamfetamine	1 (2.2)
Genitourinary system and sex hormones		2 (4.4)
G03A	Hormonal contraceptives for systemic use: dienogest; estradiol valerate, drospirenone; ethynyl estradiol	2 (4.4)
**ATC Classification**		**n (%)**
Sensory organs		2 (4.4)
S01A	Anti-infectious agents: besifloxacin	1 (2.2)
S01G	Decongestants and anti-allergy agents: alcaftadine	1 (2.2)
Dermatological agents		1 (2.2)
D06A	Antibiotics for topical use: retapamulin	1 (2.2)
Musculoskeletal system		1 (2.2)
M05B	Drugs that affect bone structure and mineralization: denosumab	1 (2.2)
Blood and hematopoietic organs		1 (2.2)
B02B	Vitamin K and other hemostatics: eltrombopag olamine	1 (2.2)
Various		1 (2.2)
V03A	All other therapeutic products: sugammadex	1 (2.2)
Total		46 (100.0)

In the package insert of 40 (87%) of the new medications, there were indications for children, and the specific age group for which it was intended was included. In 6 of the 46 medications with pediatric indication, merely “pediatric use” was included; these were classified as indicated for all age ranges, including premature and full-term newborns. No medication was identified with explicit indications for neonates. The age ranges with the smallest proportions of pediatric indications were pre-school, school, and adolescent ( Table 2 ).

**Table 2:** Distribution of 46 medications registered with pediatric indication abroad and of 37 medications registered with pediatric indication in Brazil

Age group	Abroad n=46	Brazil n=37
	n (%)	n (%)
Stratification		
Premature newborn	3 (6.5)	4 (10.8)
Full-term newborn	3 (6.5)	4 (10.8)
Infant	16 (34.8)	8 (21.6)
Pre-school	27 (58.7)	21 (56.7)
School	37 (80.4)	30 (81.1)
Adolescent	46 (100.0)	37 (100.0)

Among the 46 medications with pediatric indication, one was marketed in two different pharmaceutical specialties, one for nasal use, and another for pulmonary use. Considering 47 pharmaceutical specialties, we noted that 22 (46.8%) were intended for oral administration, 12 (25.5%) for intravenous, 7 (14.9%) for subcutaneous, 2 (4.3%) for nasal, 2 (4.3%) for eye use, 1 (2.1%) for dermal, and 1 (2.1%) for pulmonary use.

As to the dosage form of 47 pharmaceutical specialties, the most prevalent were solid for oral use (42.5%) and parenteral (40.4%), as shown on table 3 . Among 20 pharmaceutical specialties that were in solid form, the predominance of tablets and capsules was highlighted, and there was only one dispersible tablet, and one chewable. Two specialties in liquid form for oral use were observed.

**Table 3:** Dosage form of 46 medications registered with pediatric indication abroad and marketed in Brazil, from 2003 to 2013

Dosage form	n (%)
Solid dosage form	20 (42.5)
Tablet	12 (25.5)
Capsule	6 (12.8)
Chewable tablet	1 (2.1)
Dispersible tablet	1 (2.1)
Parenteral dosage form	19 (40.4)
Intravenous parenteral	12 (25.5)
Subcutaneous parenteral	7 (14.9)
Inhaled dosage form*	3 (6.4)
Dry powder device for inhalation	1 (2.1)
Nasal spray	2 (4.3)
Liquid oral dosage form	2 (4.3)
Ophthalmologic solution	2 (4.3)
Ointment	1 (2.1)
Total	47 (100.0)

* One medication presents two different dosage forms.

**Figure 1:**
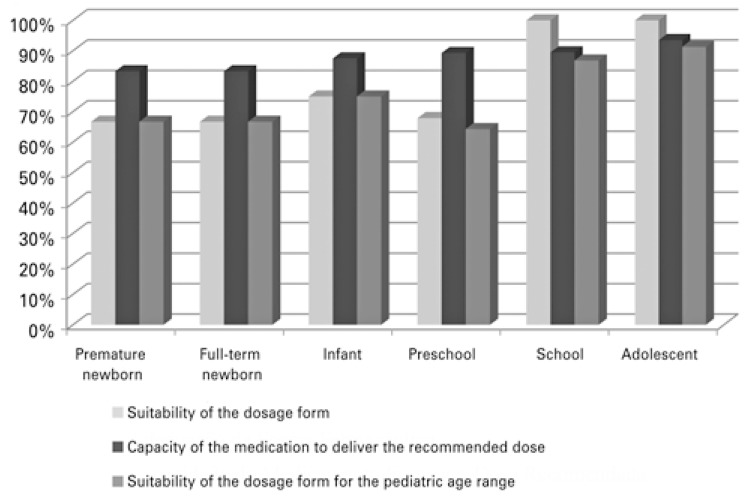
Suitability of the dosage form of 46 medications with pediatric indication

Suitability due to the capacity of the medication to provide the dose recommended was higher than 80% in all age ranges ( Figure 1 ). As to suitability of the dosage form, we identified that the greater the age range, the greater the suitability for pediatric use. Among 20 medications in soliddosage form, 10 (50.0%) were classified as suitable for all pediatric age ranges for which they were registered. In the assessment of the capacity to deliver the recommended dose, 16 (80.0%) were identified as being suitable; the four (20%) cases of inadequacy were medications with dosage prescribed as mg/kg/day. The suitability rate of the medications sold in the parenteral, liquid for oral use, topical, and inhaled dosage form was 100% in two criteria analyzed. In the analysis of suitability of the medications, the pediatric age range presented in figure 1 shows that suitability was lower for preschool age (64.3%), premature newborns (66.7%), and full-term newborns (66.7%).

The pharmaceutical excipients with potential to cause damage to children were identified in 22 (46.8%) of 47 pharmaceutical specialties, in which, in 3 specialties, more than one excipient was identified. Among the 25 occurrences of excipients, 12 (48.0%) corresponded to polysorbate 80, 7 (28%) to lactose, 3(12%) to benzalkonium chloride, 2 (8.0%) to propylene glycol, and 1 (4.0%) to ethanol. Among 22 specialties presenting excipients with potential to cause harm in children, 9 (40.9%) were for parenteral use, 8 (36.4%) solid for oral use, and 5 (22.7%) accounted for the remainingdosage forms.

## DISCUSSION

During the period from 2003 to 2013, the number of new drugs registered in Brazil for the pediatric population was small, since about one third of the drugs registered for children less than 6 years of age were classified as being unsuitable for the pediatric age range intended. In addition, it was also noted that the lower the age of the child, the lower the number of new pediatric medications registered in the country. These characteristics of the Brazilian market of medications is similar to that described in the United States and Europe.^(^ ^8^ ^,^ ^25^ ^,^ ^26^ ^)^ Thus, we see the existence of a gap in the ability to adjust the availability of medications destined for pediatric use, in the same proportion as they are offered for adult use on the market today. This can be attributed to the complexity of formulating medications for children who have the specificities of the different phases of childhood.^(^ ^1^ ^,^ ^26^ ^)^ One can see that children still occupy the status of therapeutic orphans, especially in the lower age ranges, since there is a deficit of pediatric medications. Despite the fact that pharmaceutical industries have promoted great advances on the theme, allied with the recent changes in legislation carried out especially by the regulating agencies of the United States and European Union, seeking to widen the availability of medications suitable for children, there is still the situation of therapeutic orphans.^(^ ^7^ ^)^ The lack of drugs for neonatology launched during that period, besides the reduced number of drugs for pediatric cancer and to treat bacterial infections, makes this fact a concern and shows that the criteria of pharmaceutical industries for launching drugs do not fully take into consideration the needs of children.^(^ ^26^ ^)^

This deficit of medications with pediatric indication is a worldwide reality, which is not different in Brazil;^(^ ^12^ ^,^ ^27^ ^)^ consequently, it may threaten patient’s safety by increasing the risk of adverse events due to the use of off label or non-licensed medications.^(^ ^27^ ^)^

Liquid dosage forms are considered by healthcare professionals as the most appropriate for the treatment of children, since they display ease in administration and swallowing, and greater flexibility for therapeutic dose adjustment, besides allowing better adherence of children with treatment.^(^ ^5^ ^)^ Even so, oral liquid dosage form show problems with palatability, chemical, physical, an/or microbiological stability, and risks of dose measurement errors, which is a challenge for the development of pediatric medications.^(^ ^1^ ^,^ ^10^ ^)^ This challenge can explain the reduced number of these formulations among the medications studied.

In light of the challenges for the development of liquid dosage forms appropriate for children, in 2008, the WHO proposed that the flexible solid oral dosage forms be considered the preferred formulations for children. They correspond to the solid dosage forms that do not need to be ingested whole, such as, for example, dispersible, effervescent, and oro-dispersible tablets. For greater access of children to the flexible solid dosage forms, both in developing and developed countries, as well as in poor countries, the WHO fosters implementing technological platforms for research and production of such medications. Despite the technical and economic advantages, besides ease in administration, the acceptance of these dosage form can be influenced by cultural factors. In this sense, the WHO seeks to increase awareness of children’s parents and caregivers as to the benefits of the flexible solid oral dosage forms. Another aspect intended for improvement of acceptance by children and that is being addressed is the improvement of palatability of the formulations.^(^ ^28^ ^)^

Among the new medications registered during the period of the study, the proportion of medications in flexible solid oral dosage forms was greatly decreased, showing the relevance of WHO actions in widening access to these dosage forms. The solid forms were prevalent in the cohort investigated and contributed significantly to the classification of medications as inadequate for the pediatric age range, since only one presented with the capacity to provide flexible doses, since it is a dispersible tablet. It is important to point out that the most frequent dosage regimes for solid medications studied were in number of units of the solid form to be administered. If medications with doses expressed in mg/kg/day were greater in number, a greater proportion of unsuitability would have been found.

In order to make available safe and effective oral use medications for children, technological innovations are being proposed, such as mini tablets, oro-dispersible coatings, and liquid formulations based on milk.^(^ ^10^ ^)^ Studies with mini tablets are in an advanced phase, and the results showed that those measuring 2mm can be given to infants and premature neonates; those with 4mm are suitable for children older than one year of age.^(^ ^10^ ^,^ ^11^ ^)^ Acceptance of the mini tablets by children was superior when compared with the acceptance of powder for oral use, syrup, suspension, and solution.^(^ ^10^ ^)^ Another advantage of mini tablets is the possibility for design the modified release dosage form^(^ ^1^ ^-^ ^10^ ^)^ which can reduce the number of doses given and ease adherence. The EMA guidelines, which orient the development of medications, established that the mini tablet could be considered a measure to increase acceptability and flexibility of doses for children.^(^ ^10^ ^)^

The pharmaceutical excipients, known for lacking pharmacological action, represent another important factor to be considered in the development of medications for children. The influence of excipients in the safety profile of the pediatric medication has been described, due to immaturity of organs and body systems of the child. Excipients that are safe for adults may not be safe for children of lower ages.^(^ ^1^ ^,^ ^10^ ^)^ The concern is growing with the presence of excipients in pediatric medications, such as polysorbate 80, which showed a high frequency among the medications studied.^(^ ^10^ ^,^ ^21^ ^-^ ^23^ ^)^ Even so, a clearer definition of excipients with greater potentiality to cause harm only exists for neonates.^(^ ^23^ ^,^ ^29^ ^)^

The influence of polysorbate 80 on the activation of glycoprotein P and the potential to induce thrombocytopenia, renal dysfunction, and metabolic acidosis, calls attention to the risks of using pediatric medications, especially in neonates and smaller children.^(^ ^24^ ^)^

The excipients selected for potentially causing harm to children were found in almost half of the medications studied, which demands a warning as to the dimension of the problem. However, evaluation of the suitability of the medication to the age range relative to the pharmaceutical excipients cannot be considered absolute, since a limited number of excipients was chosen; the safety evaluation of an excipient in a pediatric formulation depends on the target age group and on additional information, such as the maximum concentration and dose allowed for daily ingestion.^(^ ^10^ ^)^ This information is not available on Brazilian package inserts. Therefore, the evaluation of suitability of the medication to the pediatric age ranges in this study covered only the dosage form and the capacity to deliver a suitable dose.

Different from the United States and European countries, Brazil does not have specific legislation that regulates and encourages the registration of pediatric medications.^(^ ^12^ ^)^ Nevertheless, the Ministry of Health, in light of the problematic issues that involve the use of medications by children, organized a working group to foster the creation of public policies directed towards the improvement of pediatric pharmacological therapeutics. In 2017, the document “ *Assistência Farmacêutica em Pediatria do Brasil – Recomendações e estratégias para a ampliação da oferta do acesso and do uso racional de medicamentos em crianças”* [Pediatric Pharmaceutical Services in Brazil - Recommendations and strategies for widening the offer of access and rational use of medications in children], which presents widespread actions proposed by the working group to change the national panorama of difficulties in child care and consequently, to improve pediatric therapeutics.^(^ ^27^ ^)^ This initiative of the Brazilian government is compliant with the WHO ‘Made Medicines for Children Size’ project, that encourages countries to implement actions in order to broaden the availability of medications suitable for children.^(^ ^30^ ^)^

This study brought significant contributions to the knowledge of suitability for the pediatric age ranges of the new drugs registered in Brazil, from 2003 to 2013. Additionally, it is innovative, since research with new medications registered in Brazil are prior to 2003, and do not focus on the child’s perspective.^(^ ^27^ ^)^ However, it is important to point out as limitation of the study the fact that the evaluation of the pediatric medication was restricted to the pharmacotechnical aspects. Other factors aiming to contribute to parents, caregivers, and nursing professionals administering medications with safety, such as the nature of the device for measuring doses, stability of the formulation, and the instructions for use of the medication, were not assessed. Palatability, which is important for the acceptance of the medication by the child and relevant to promote adherence with treatment, was neither evaluated. Absence of the evaluation of these factors did not allow one to know the entire dimension of suitability of medications for pediatric use. Additionally, the analysis was restricted to the medications marketed in 2016, due to the need to gather information relative to the excipients of the formulation, which are in the package insert, but are not available in the ANVISA database.

## CONCLUSION

Medications destined for children aged under 6 years presented with a lower frequency of suitability, considering the dosage form and capacity to provide the dose recommended. Availability and suitability of the medications for pediatric use increased with older age ranges for which the medication is registered. The frequency of medications with the presence of excipients in the formulation capable of causing damage to children was high.
